# Has Radiotherapy Been Successfully Implemented in Alberta’s Small Cities? A Review of Alberta’s Regional Cancer Centre Network from 2010–2020

**DOI:** 10.3390/curroncol28010047

**Published:** 2021-01-13

**Authors:** Peter S. Craighead, Dean Ruether, Chandra Martens, Petra Grendarova

**Affiliations:** 1 Tom Baker Cancer Centre, Calgary, AB T2N 4N2, Canada; 2 Cumming School of Medicine, University of Calgary, Calgary, AB T2N 1N4, Canada; dean.ruether@ahs.ca; 3 Cancer Care Alberta, Alberta Health Services, Edmonton, AB T5J 3E4, Canada; 4 Central Alberta Cancer Centre, Red Deer, AB T4N 6R2, Canada; chandra.martens@ahs.ca; 5 Grande Prairie Cancer Centre, Grande Prairie, AB T8V 2E8, Canada; petra.grendarova@ahs.ca

**Keywords:** care close to home, distributed radiotherapy, community cancer care

## Abstract

The expansion of cancer services closer to home has become a major focus of publicly funded healthcare, with cancer organizations attempting to invest in smaller centers by integrating radiotherapy into these facilities. In Canada this has resulted in Ontario, British Columbia and Alberta investing in 12 expanded regional centers over the past 20 years. Quebec, Manitoba and Nova Scotia have made similar investments. Alberta’s three new centers opened in 2010, 2013 and 2021 (projected). This study examined improvements in wait times and patient throughput between 2010 and 2020, and highlighted strategies that will support the sustainability and growth of clinical activity through to 2030. Significant improvement in ready to treat wait times for radiotherapy have resulted from opening two centers, and the provincial throughput for patients requiring systemic or radiotherapy has gone up by 16%. A patient satisfaction survey demonstrated that rural patients are happy with their care and desire the provision of more of their cancer treatment closer to home. An expert panel provided recommendations on what needs to be done to stabilize recruitment and retention.

## 1. Introduction

In 2006 the Canadian federal government announced an initiative to improve national wait times in health care, with the assumption that their provincial counterparts would provide an equivalent investment to address wait times issues [1]. In Alberta this enabled the incumbent government to focus its attention on reducing wait times for radiotherapy, resulting in a “corridor” plan. Its major outcome was the establishment of expanded regional cancer centers in three cities (Lethbridge, Red Deer and Grande Prairie) by 2020 [2]. Together with the tertiary centers, these five centers were anticipated to manage the workload capacity and improve wait times/increase care close to home. Since 2010 the plan has resulted in new centers in Lethbridge (JACC) and Red Deer (CACC), and one will open in Grande Prairie (GPCC) in 2021. Radiotherapy and comprehensive systemic treatment services will be provided close to home to most Albertans across five sites by 2021.

This study follows one published by Craighead et al. in Current Oncology in 2011, which looked at the elements required for smaller cities to successfully implement radiotherapy [3]. It suggested that the following drivers were critical for successful implementation: a tangible connection between regional and tertiary centers; selecting cities with a sufficient population to justify two linear accelerator departments; and ensuring manpower issues were proactively addressed. The current study arose from a service plan project that examined the strategies for growth till 2030, and evaluated whether the capital investment in these three cities had resulted in the successful implementation of radiotherapy. It also looked at the challenges resulting from efforts to increase the proportion of patients receiving care closer to home.

## 2. Experimental Section

### 2.1. Methods

This is a mixed methods study, using qualitative data to compare the provision of radiotherapy and systemic treatment services in Alberta between 2010 and 2020. It also used a qualitative component to review barriers to growth and to recommend strategies to mitigate these. Prior to 2010 radiotherapy was not delivered outside the larger cities, Calgary and Edmonton. The objectives of the study were to evaluate:

1. Ready to treat (RTT) wait times*. The study aimed to confirm that Albertans requiring radiotherapy in 2009 before small centers were opened had experienced improvement in wait times by 2020. (*Ready to treat wait time is the time between the date when a patient is ready for referral for treatment, and the date when treatment itself starts.);
2. Proportion of patients undergoing treatment in the smaller centers. It was believed that by 2017 we would see that more than 80% of patients who resided in the regional cancer center catchment areas with breast/gastrointestinal (GI)/genito-urinary (GU) and lung cancer to have received their radiotherapy and systemic treatment locally (2017 was the latest audited data available). They consulted a radiation oncologist (RO) prior to therapy;
3. Patient satisfaction. It was hoped that more than 80% of patients receiving radiotherapy/systemic therapy in regional centers would report high levels of satisfaction;
4. Barriers to close to home care. An expert panel was established from internal leaders in the province to provide insights into how to improve care close to home. They reviewed reports from interviews of regional center leadership, plus held discussions with executive leaders.

### 2.2. Materials

The study used data from the Clinical Data Integration unit and from the 2010 Alberta Radiotherapy corridor planning (ACP) document to compare 2009 and 2020 provincial wait times for radiation therapy. The 2009 time period was used because it was the last year prior to opening the first small radiotherapy department, and confidence in the accuracy of wait time data prior to 2009 was not high. The Alberta Cancer registry and Clinical Data Integration unit provided data on the spectrum of patients seen and treated in regional centers in 2017 (the most complete data), and the Canproj method was used to project patient numbers to 2030. Canproj has been shown to accurately predict for growth in patient numbers by integrating ageing and prevalence into projections. This last analysis simply allowed us to predict service growth for the next decade.

A patient satisfaction survey, conducted in early November 2019, targeted all patients from outside Calgary and Edmonton, who visited a regional or tertiary center for consultation, follow up or treatment. The objective was to examine satisfaction with care across Alberta.

A qualitative research component looked at the barriers for growth in smaller centers. In an attempt to understand and address the barriers for growth, we embarked on a qualitative study to look at issues identified during interviews with leaders at all regional centers. We established an expert panel to view these interviews and provide advice on how they would address the concerns expressed. This component of the study was not rigorous, and can only provide suggested barriers and solutions. The manuscript describes the qualitative and quantitative elements associated with provision of care at a regional center, and uses an expert panel to provide insights into how to move ahead. Much of the focus is on radiotherapy services, which was the major change to small centers from 2010.

## 3. Results

Expanded centers in Lethbridge (JACC) and Red Deer (CACC) have been open for 10 and 7 years, respectively, with the provision of radiotherapy being the major change initiated by expansion. The delay in opening an expanded regional center in Grande Prairie (GPCC) by 2020 is outside the mandate of this manuscript, but once this has opened it will make even further improvements to providing care close to home. The current mandate for regional cancer centers has been to consult and treat big four cancer patients (Breast/GI/GU/Lung), and to offer palliative treatment to any patient. By opening JACC and CACC the capacity to increase the throughput of patients close to home has been increased significantly, with 9000 Radiation Oncology new consults having been provided in these two sites over the past 10 years. The number of treatment fractions provided closer to home in the last decade is about 130,000. Although this expansion has also resulted in increased systemic therapy patients, the major change has been in the way RT is provided.

Has the opening of radiotherapy facilities in two small cities helped address clinical issues across Alberta?

### 3.1. Consult and Ready to Treat (RTT) Wait Times for Radiotherapy in Alberta

ACP documents from 2010 showed that 85% of 8000 Albertans treated in 2009 started radiotherapy within 4.5 weeks of their ready to treat date, with 15% having to wait between 4.6 and 8 weeks. In March 2020, 98% of patients started radiotherapy within 4 weeks of their RTT date, with 64% having started within 2 weeks (this is a consistent wait time that has been maintained for over 2 years). This significant improvement (*p* < 0.001 *t*-test) in RTT times occurred in spite of a 16% increase in new patient consults provincially between 2010 and 2020, with the number of newly treated patients projected to be 9200 cases for 2020 based on the prorated caseload till September 2020. The increase in overall numbers seen, and improved wait times, is directly related to the increased capacity created by the opening of two new centers.

### 3.2. Spectrum of Patients Receiving Radiotherapy or Systemic Treatment in Smaller Cities

Treatment data were analyzed relative to a close to home (CCTH) parameter. This measure is realized by summating new patient treatments relative to where they live (municipality or county location), and whether treatment had occurred at the cancer center that was closest to them. This was calculated in a proportional format, because this was the measure that our government had demanded we use (and has been provided to them for the past decade). The data for systemic treatment in Grande Prairie were examined, but treatment data for RT is impossible since their department will only open in 2021. There is consultation data from GPCC because of a consultant traveling there monthly. This close to home parameter allowed us to demonstrate the following.

#### 3.2.1. Radiotherapy for the Big 4 Patients in Lethbridge/Red Deer and Grande Prairie

In 2010 it was agreed that smaller centers would have a mandate to consult and treat at least 80% of local patients with big 4 cancers, as well as those needing palliative treatment. Data from 2017 show that the proportion of patients consulted or treated locally with the big 4 cancers varied significantly across the three small regional centers (see Table 1). Between 54 and 78% of these local patients underwent consultation and treat close to home (CCTH). When we examine the proportion of all cancer patients (not just the big 4) receiving consultation near their closest center, only 42 to 63% of local patients underwent consultation. As regards treatment, 85% of the big 4 patients (765 of 800) were treated close to home at JACC or CACC in 2017. Grande Prairie’s radiotherapy treatment numbers cannot be compared because their RT department only opens in 2021.

#### 3.2.2. Systemic Treatment for the “Big 4” Tumor Sites

Consultation and systemic treatment occurred in higher proportions than radiotherapy at regional centers (see Table 2), because systemic services at these centers preceded RT by 20 years. Between 81 and 84% of the big 4 patients were consulted for systemic treatment locally, with 84 to 93% receiving treatment locally. Consultation for the systemic therapy of all local cancer patients is expected to be higher than radiotherapy numbers. Because of Medical Oncology (MO) staff shortages, this was not seen, and 40% of all local diagnoses were consulted at tertiary centers, with 17 to 35% of them also receiving treatment there. This improves to over 85% for local consultation and treatment when staffing is optimal, shown by data from 2016.

#### 3.2.3. Growth in Patient Numbers by 2030 (Canproj Method)

Projections until 2030 (Table 3) show there will be increases of between 30 and 40% over the next 10 years. The complexity and chronicity of care is also expected to increase. Alberta’s registry has shown a close approximation between actual numbers seen and growth over a period of time when using Canproj. Given that most cancer centers will see 80% of newly diagnosed cases, this chart simply provides further reasons for sustaining regional cancer centers.

### 3.3. Patient Satisfaction

Five hundred and sixty patients were surveyed in November 2019, 42% of whom live more than 100 km from their closest regional/tertiary center. Approximately 90% of patients endorsed excellent care, whether it occurred in a tertiary or regional center.

- Twenty three percent (23%) stated that they were referred to a tertiary center because their referring physician felt more comfortable with care options there. This likely reflects ingrained patterns of behavior amongst referring physicians. It will require a concerted effort to educate and change the perception of some referring physicians.
- Four hundred and seventy six of the five hundred and sixty patients (85%) indicated they were willing to go anywhere for treatment, as long as the quality was equivalent.
- Patients’ overwhelming preference was to receive supportive care at regional rather than tertiary centers (64 versus 25%). Some patients were not interested in supportive care services, regardless of location.
- Fifty percent (50%) or more of local patients would be happy to have at least one of their follow up visits using telehealth; this was especially true of patients living in remote areas.

### 3.4. Qualitative Aspects of the Study

Barriers to service provision were identified by interviewing regional centers and then discussing with an expert panel. The same interview questions were asked of local leadership, and responses recorded. After four meetings with an expert panel, the following barriers to sustainable growth were identified:

1. Unfortunately, several physicians in rural towns and cities continue to refer patients to larger centers. When approached these physicians cite patient preference reasons, though we know this is not the case based on our satisfaction survey. This is compromising opportunities for care closer to home. This is especially important given that patients said that they would go wherever they were sent, as long as quality would not be compromised;
2. Absence of local medical leadership in regional centers. Alberta Health Services has embraced a centralized model of medical leadership, while endorsing a local manager model for operations. This has resulted in physicians within regional cancer centers feeling disenfranchised, and places inappropriate responsibility on local operational managers. A similar phenomenon has influenced Medical Physicists;
3. The recruitment and retention of Oncologists in regional centers between 2010 and 2020 has been challenging, especially for Medical Oncology. Eight of the thirteen Medical Oncologists recruited to work in Medicine Hat/Lethbridge, Red Deer and Grande Prairie during this time have retired or resigned, and recruitment into vacancies has taken between 6 and 18 months. There are less challenges in the retention of ROs. The challenges in retention relates to several elements, as follows:
  - Lack of support during contingency crisis. When a small center loses staff, this has a major impact on the remaining physician work force. Losing one physician in a department of two or three individuals results in a dramatic increase in work for the remaining members. Additionally, this results in tertiary centers losing efficiencies by being forced to send their staff to smaller centers. Small centers do not feel supported to deal with contingencies;
  - Smaller center oncologists are expected to carry heavier caseloads than their tertiary counterparts, because it is assumed that individuals in these locations are non-academic;
  - There is significant dependency on the recruitment of foreign trained oncologists, who require practice readiness assessments and supervised training as they pursue licensure and practice independence, a process which adds expense and a minimum of 6 months to the recruitment process.
4. Insufficient resources for supportive care. Models of supportive care have evolved significantly during the past decade, but significant gaps in expertise at regional centers remain. Comprehensive cancer care must include access to a wider spectrum of expertise, including nutritional and rehabilitation services, occupational therapy, psychosocial oncologists, social workers, speech language pathologists as well as palliative care experts;
5. Inability to provide services to patients with less common cancers. Highly specialized, resource-intensive radiotherapy treatment, requiring additional training and expertise, will remain under the purview of tertiary centers. Presently, this has included brachytherapy, pediatric radiotherapy, and some stereotactic radiotherapy techniques. Some systemic therapy options, such as bone marrow transplantation and CARR-t therapy, are also not appropriate for delivery in regional centers.

### 3.5. Qualitative Study: Experty Panel Recommendations on How to Address above Barriers

Strategies to address the barriers were identified and should be addressed if regional centers are to grow their capacity to address workload challenges by 2030. These include the following issues.

#### 3.5.1. Focus on Care Close to Home—Move Appropriate Referrals to Regional Centers Away from Over-Capacity Tertiary Centers

In spite of local oncologic expertise and treatment capacity, a significant number of new cases continue to be referred into the two tertiary centers and need to be redirected back to the appropriate regional center. Education of the referring physician community is key to changing behavior and supporting this effort, and a repatriation strategy within the tertiary cancer centers is critical in increasing the proportion of patients seen and treated in Lethbridge, Medicine Hat, Red Deer and Grande Prairie.

#### 3.5.2. Improve Physician Morale by Increasing the Retention of Physicians at Regional Centers through the Development of Regional Medical Leaders, Improvement in Manpower models, and Increased Opportunities for Academic Contribution

Working as an oncologist in a regional cancer center needs to be seen as an attractive and fulfilling alternative to working in a tertiary facility. Improved job satisfaction, potential for an individual’s growth and advancement, and academic productivity are critical to the recruitment and retention of physicians. In support of this, the following must occur:

- Regional center leaders and their physicians must be supported to advocate for their needs and provide greater influence in the cancer system;
- Manpower models must address workload issues at regional centers, creating the necessary time for physicians to contribute academically and achieve work–life balance;
- A stronger academic relationship between regional oncologists and tertiary centers will improve the opportunities for the advancement of oncologists in the regional centers.

#### 3.5.3. Build Robust Non-Specialist Manpower, and Establish Innovative Service Delivery Models

Manpower must be able to address current workloads, and support the provision of the additional supportive care resources needed, especially as patient numbers grow and a broader range of patients are cared for at regional centers. Poor retention and low morale must be addressed. Heavy clinical workloads need to be reduced through innovative service delivery models (GPs GPOs, NPs, advanced therapists, clinical pharmacists), support from tertiary center-based colleagues, and an expanded capacity for virtual health care. Oncologists at regional centers need to be supported in their desire to flourish academically and benefit from opportunities to collaborate with tertiary center-based colleagues [4,5]. All of these have been integrated into service delivery models in British Columbia and Ontario.

#### 3.5.4. Phase in Consultation and Treatment of Non-Big 4 Patients Diagnosed with Cancer, Starting in Red Deer

Support the development of specialty focus within oncology at regional cancer centers. This would lead to a larger number of patients being treated locally and a broader range of patients benefiting from care closer to home. Manpower, additional training requirements and support from tertiary colleagues are identified as barriers to success in these efforts. Red Deer and the Central Zone are experiencing population growth and a sufficient volume of cases exists to justify oncologists developing expertise in sites other than breast, GI, GU and lung cancer [6]. Facilitating this is critical, given the projected growth by 2030 and the desire to support more care closer to home. Similar trends are evident in Lethbridge and the South Zone.

#### 3.5.5. Expand the Provision of Supportive Care Services in Regional Centers

Supportive care services require investment within the South, Central and North Zones. This will be especially relevant when these centers expand to treat patients with head and neck cancers, or other complex diseases. A comprehensive plan to address supportive care is being planned for 2021.

## 4. Discussion

Since 2010 Alberta has successfully implemented radiotherapy services in two smaller cities, with a third expanded regional cancer center opening in 2021. Radiation and systemic treatment will be available to Alberta cancer patients across five centers by 2021. This implementation has improved provincial RTT wait times, and allowed major cancer patients to receive their treatment close to home. The fact that an additional 9000 new cases were seen for radiotherapy between 2009 and 2020, with 8000 of them receiving treatment, supports the notion that these centers have increased access to radiotherapy in the province. The fact that over the past decade the provincial average number of fractions per patient has dropped from 16 to 14 has allowed these new centers to treat high numbers of patients. As these new facilities accept more complex patients, this throughput will level off. Regional centers have focused mainly on patients with breast, GI, GU and lung cancers. Patients are satisfied with the quality of care they are provided at our regional centers. The COVID crisis has forced Cancer Care Alberta to expand its use of virtual health technologies, with this being strongly endorsed by rural patients. It should become a standard component of how we offer care closer to home in the future.

How should Cancer Control Alberta address the challenges for the future growth of regional centers? There is no single gold standard against which Alberta can measure the effectiveness of our smaller centers. The expert panel previously alluded to looking at national benchmarks, to provide recommendations for the future [7]. This panel used benchmarks from Ontario (Cancer Care Ontario) and British Columbia (British Columbia Cancer Agency), given similar challenges such as population density, distribution of cases and the relative size of cancer centers in the three provinces. The panel provided pragmatic advice to the Cancer Care Alberta executive in May 2020.

### 4.1. Contextual Findings of Benchmark Centers in Ontario and British Columbia

Most Canadian provinces have provided systemic therapy services in a distributed way for the past 30 years, in support of care closer to home. This strategy has utilized medium-sized regional hospitals to run clinics and systemic treatment day care units, allowing patients to receive consultation at a larger center, and subsequently receive their systemic treatment in or near their home community. In Alberta, this care occurs in 11 community locations outside of Edmonton and Calgary, with four larger regional centers providing consultation and systemic treatment (Lethbridge, Medicine Hat, Red Deer and Grande Prairie). In Ontario, British Columbia and Alberta, a proportion of these regional centers have expanded to offer radiotherapy.

The integration of radiotherapy into smaller cities has been a major focus for cancer organizations in Canada, but requires approaches that are quite different from centers with systemic services alone. Decisions about where to offer radiotherapy are usually driven by having a sufficient workload to justify the presence of a minimum of two linear accelerators (between 400 and 600 new cases annually) [4,5]. Over the past 20 years this philosophy will have resulted in six new regional cancer centers being built with radiotherapy in Ontario, three new centers in British Columbia, and three new centers in Alberta by 2021 [7,8]. This is a significant investment in care close to home and reduces the demand for services in tertiary cancer centers [8]. Such investment was justified within Alberta when one considers that 28% of Alberta’s population lived more than 100 km away from Calgary and Edmonton in 2008 [9]. The delivery of care closer to home results in other indirect cost savings to cancer patients, including those associated with travel, accommodation, and absences from work. In addition, it allows patients with limited prognoses to spend more times with their loved ones.

Standards used by Ontario and British Columbia provide partial answers to steps needing to be taken by Alberta over the next decade, including the following.

#### 4.1.1. Importance of Having Local Medical Leadership, and a Stable Local Medical Team

Newly opened regional centers in Ontario and British Columbia have appointed regional medical leaders that function in a dyad partnership with operational regional vice presidents. Medical and operational leaders report to provincial heads at a central level. The model has worked well in BC and Ontario, evidenced by stability in recruitment, retention, and the delivery of quality care. Alberta’s centralized leadership model served it well between 2010 and 2020, but the challenges of recruitment and retention and the need to support local improvements and enhancements to care make it critical that Alberta consider investing in the development of local leaders. Such investment is key to creating career development opportunities and the pursuit of academic endeavors that will result in physician retention. This investment will also maximize job satisfaction in other areas. A central medical leadership model for the community cancer program will remain critical for the 11 community cancer centers and for some smaller regional centers.

The stability of local medical teams is critical for growth. Successful recruitment and retention will be strong drivers of a desirable working environment. Such an environment will include opportunities to bring advancements to the care of patients in regional centers, opportunities for personal growth, and academic productivity (including participation in research and clinical trials). Regional centers should also be involved in training the next generation of oncologists. The adoption of healthy manpower guidelines that ensure regional sites are appropriately staffed must be a priority.

#### 4.1.2. Relationship with Tertiary Sites and the Advancement of Academic Opportunities

A healthy supportive relationship between regional and tertiary center oncologists is critical. Newly trained oncologists and their patients benefit from the support of more experienced colleagues, and quality assurance at regional centers can only happen with the support of tertiary sites.

Patients and oncologists at regional centers have identified a desire for access to clinical trials, which are often a challenge to provide in smaller cancer centers. Clinical trials programs based at the tertiary sites should partner with regional center oncologists in support of this, and the infrastructure at tertiary sites must be leveraged for the regional centers and their oncologist to be successful in this regard. Such efforts would enhance the ability to accrue trials and improve the patient experience.

#### 4.1.3. Team-Based Care, Innovative Approaches

Central to the provision of excellent cancer care is the requirement for well-functioning multidisciplinary care teams. Such team-based care must include expertise from physician-based oncology disciplines and generalists, nursing and nursing navigation, radiation therapy, guideline experts, supportive care personnel, and operational managers. Rarely do cancer patients require a single modality of care that can be provided by one member of a team, and patients’ needs change over time as they move through their cancer journey. Regional centers must be adequately resourced for the provision of holistic cancer care in order to provide optimal opportunities for care closer to home.

#### 4.1.4. Repatriation of Certain Regional Patients to Regional Centers, and Increase in the Spectrum of Cases Being Seen at Regional Centers

Repatriation of the big 4 cases currently referred inappropriately to tertiary sites will be critical for further action on provincial RTT wait times and will support more patients in care closer to home. Tertiary sites are struggling to manage their workload in the face of increased referrals and the higher complexity of care. Alberta Health Services must adopt marketing strategies to encourage local physicians to use the regional facilities that provide excellent care on par with that provided at the tertiary centers. Expanding the spectrum of patients that can be treated at regional centers requires a thoughtful approach. The Central Alberta Cancer Center in Red Deer has a sufficient volume of potential patients to warrant a phased approach to the introduction of radiotherapy services to patients that are not presently part of the center’s mandate. This will require support from tertiary sites and increased opportunities for additional training.

## 5. Conclusions

In conclusion, this study was conducted to ascertain whether or not the financial investment in regional centers in Alberta had made improvements in caring for patients closer to home. It also examined the strategies needed to move ahead and address the projected cancer case growth by 2030. The study has demonstrated that the opening of two new regional centers in Lethbridge and Red Deer has improved provincial wait times for radiotherapy. It also demonstrated that patients are satisfied with their care wherever this is provided, and that these new centers have resulted in a high proportion of the big 4 patients receiving both systemic and radiotherapy locally. This will improve further with the opening of an expanded center in Grande Prairie (2021).

There are several challenges to maintaining growth in patient numbers, and to increasing care close to home opportunities, as described by the expert panel. This study provides some strategies to address those challenges.

## Figures and Tables

**Table 1:** New consults and treatments for radiotherapy according to CCTH (consultation and treat close to home) in 2017.

Regional Center	RO New Consults		Radiation Therapy	
	Breast, Lung, GI, GU pts. only	All Tumor Types	Breast, Lung, GI, GU only	All Tumor Types
**JACC**	75%	62%	81%	63%
**CACC**	78%	63%	88%	71%
**GPCC**	54%	42%	N/A	N/A

RO-radiation oncologist, GI- gastrointestinal, GU-genito-urinary, JACC-expanded regional center in Lethbridge, CACC-expanded regional center in Red Deer, GPCC-expanded regional center in Grande Prairie.

**Table 2:** New consults and treatments for systemic therapy according to CCTH in 2017.

Regional Center	MO Consults		Systemic Therapy	
	Breast, Lung, GI, GU only	All Tumor Types	Breast, Lung, GI, GU only	All Tumor Types
**JACC**	84%	60%	93%	83%
**CACC**	81%	60%	91%	60%
**GPCC**	81%	62%	84%	71%

MO-medical oncology staff.

**Table 3:** 10-year growth projection for new cancer cases in Alberta’s three regional centers.

Tumor Types	JACC				CACC		GPCC		
Year	2020	2025	2030	2020	2025	2030	2020	2025	2030
Current Mandate	1142	1245	1356	1258	1441	1619	577	644	716
Endocrine	24	27	29	43	48	52	25	28	31
Head and Neck	46	50	54	48	52	57	24	26	29
Hematology	196	218	240	194	219	244	94	106	118
Melanoma	68	78	88	69	80	90	20	22	24
Gynecology	95	105	114	96	107	118	47	53	59
Other Cancers	63	66	69	70	76	85	33	35	36
Total	1635	1789	1950	1777	2023	2265	820	914	1013
% increase from 2017	9%	19%	30%	7%	22%	36%	14%	27%	41%

## Data Availability

No new data were created or analyzed in this study. Data sharing is not applicable to this article.
